# The association between proton pump inhibitors and the risk of gastrointestinal bleeding in oral anticoagulants users

**DOI:** 10.1038/s44325-024-00037-3

**Published:** 2025-04-12

**Authors:** Zixuan Wang, Qiuyan Yu, Charlotte Warren-Gash, Krishnan Bhaskaran, Clémence Leyrat, Ka Shing Cheung, Celine S. L. Chui, Esther W. Chan, Ian C. K. Wong, Amitava Banerjee, Liam Smeeth, Ian J. Douglas, Angel Y. S. Wong

**Affiliations:** 1 Faculty of Epidemiology and Population Health, London School of Hygiene and Tropical Medicine, London, United Kingdom; 2 Laboratory of Data Discovery for Health (D24H), Hong Kong Science and Technology Park, Hong Kong, China; 3 School of Pharmacy, School of Health Sciences, College of Medicine and Health, University of Birmingham, Birmingham, United Kingdom; 4 Centre for Safe Medication Practice and Research, Department of Pharmacology and Pharmacy, The University of Hong Kong, Hong Kong, China; 5 Department of Medicine, School of Clinical Medicine, Queen Mary Hospital, The University of Hong Kong, Hong Kong, China; 6 Department of Medicine, The University of Hong Kong-Shenzhen Hospital, Shenzhen, China; 7 School of Nursing, Li Ka Shing Faculty of Medicine, The University of Hong Kong, Hong Kong, China; 8 School of Public Health, Li Ka Shing Faculty of Medicine, The University of Hong Kong, Hong Kong, China; 9 Aston School of Pharmacy, Aston University, Birmingham, United Kingdom; 10 Institute of Health Informatics, Faculty of Population Health Sciences, University College London UCL, London, United Kingdom

**Keywords:** Cardiovascular diseases, Health care

## Abstract

Current evidence of whether proton pump inhibitor (PPI) reduces the risk of gastrointestinal bleeding (GIB) associated with oral anticoagulants (OACs) is limited. Propensity score-weighted cohort and case-crossover studies were conducted separately in England and Hong Kong between 2011.01.01 and 2019.12.31. In the cohort design, we compared the hazards of hospitalised GIB in ***OAC*** + ***PPI*** users with ***OAC only*** users in people with atrial fibrillation and found higher hazard of GIB in ***OAC*** + ***PPI*** users in both settings. In the case-crossover design, elevated odds of exposure to ***PPI only***, ***OAC only*** and ***OAC*** + ***PPI*** associated with GIB between 30-day hazard and referent periods were similarly found in both settings. Overall, the evidence of an elevated risk of ***OAC*** + ***PPI*** associated with GIB compared with ***OAC only*** was modest in the cohort study. Our case-crossover study suggested that residual confounding is likely to explain the association, suggesting that concomitant prescription of PPI with OAC did not modify GIB.

## Introduction

Oral anticoagulants (OACs) including direct oral anticoagulants (DOACs, *dabigatran, rivaroxaban, apixaban and edoxaban*) and the older alternative, *warfarin*, are commonly used for the prevention of arterial embolism among patients with atrial fibrillation (AF) and the treatment and prevention of venous thromboembolism (VTE)^1^. However, the common side effect of taking OACs is bleeding^2^.

Some studies have suggested that proton pump inhibitors (PPIs) might reduce the risk of gastrointestinal bleeding (GIB) associated with OACs by addressing the mechanisms of GI mucosa protection and gastric acid reduction^3–5^. However, some showed contradictory findings as PPIs may interact with OACs, leading to potential alterations in drug activity and absorption^6,7^. Data are also lacking to understand the impact of PPIs on the risk of GIB by individual DOAC. Further, a review reported the ethnic differences in the risk of GIB in people taking OACs^8^. In general, Asians have lower hypercoagulability than Caucasians, leading to a lower risk of thrombosis and an increased risk of bleeding compared with Caucasians^9,10^. The pharmacokinetics of different DOACs could also be different between Asians and Caucasians^11–14^. Thus, it is important to understand the role of PPIs in OAC users among Asians and non-Asians and whether the risk of GIB varies across different DOACs.

Therefore, this study aimed to investigate the risk of GIB associated with the combined use of OAC and PPIs compared with OAC only in people with AF using routine clinical data in England and Hong Kong. We separately conducted the analysis for warfarin and DOACs.

## Results

In the study period of 2011–2019 inclusive, we identified 76,156 people prescribed ***warfarin*** + ***PPI***, 69,456 prescribed ***warfarin only***, 87,743 prescribed ***DOAC*** + ***PPI***, and 110,096 prescribed ***DOAC only*** in both settings. Tables 1–2 show the baseline characteristics of these exposure groups. After PS weighting, good covariate balance was attained with all standardised mean differences <0.05 in both settings (Supplementary Table 1).

**Table 1:** Baseline characteristics in CPRD Aurum before propensity score weighting

	Warfarin + PPI	Warfarin only	DOAC + PPI	DOAC only
Total	67,665	61,092	73,029	93,448
Age at index date				
Median (IQR)	77.4 (70.3–83.2)	75.6 (67.6–82.0)	77.8 (70.4–84.3)	76.2 (68.3–83.5)
Min, max	20.1, 104.3	18.3, 105.9	21.2, 106.0	18.5, 105.4
Age group				
18– < 40	198 (0.3)	419 (0.7)	161 (0.2)	572 (0.6)
40– < 50	813 (1.2)	1465 (2.4)	771 (1.1)	1930 (2.1)
50– < 60	3317 (4.9)	4577 (7.5)	3804 (5.2)	6814 (7.3)
60– < 70	11,995 (17.7)	12,878 (21.1)	12,537 (17.2)	18,638 (19.9)
70– < 80	25,281 (37.4)	21,881 (35.8)	25,439 (34.8)	30,845 (33.0)
80 +	26,061 (38.5)	19,872 (32.5)	30,317 (41.5)	34,649 (37.1)
Male sex	37,816 (55.9)	34,696 (56.8)	38,776 (53.1)	52,272 (55.9)
Calendar year at cohort entry				
2011	10,167 (15.0)	11,005 (18.0)	31 (0.0)	64 (0.1)
2012	11,450 (16.9)	11,847 (19.4)	625 (0.9)	1002 (1.1)
2013	11,245 (16.6)	11,742 (19.2)	1955 (2.7)	3060 (3.3)
2014	10,704 (15.8)	10,258 (16.8)	4217 (5.8)	6400 (6.8)
2015	8829 (13.0)	7638 (12.5)	8378 (11.5)	12,196 (13.1)
2016	5865 (8.7)	4167 (6.8)	11,533 (15.8)	15,733 (16.8)
2017	4054 (6.0)	2169 (3.6)	13,826 (18.9)	17,699 (18.9)
2018	2764 (4.1)	1248 (2.0)	15,030 (20.6)	18,661 (20.0)
2019	2587 (3.8)	1018 (1.7)	17,434 (23.9)	18,633 (19.9)
Body mass index				
Underweight	1183 (1.7)	952 (1.6)	1605 (2.2)	1975 (2.1)
Normal	17,758 (26.2)	16,411 (26.9)	19,295 (26.4)	26,412 (28.3)
Overweight	24,311 (35.9)	22,028 (36.1)	25,700 (35.2)	33,030 (35.3)
Obese	23,381 (34.6)	20,588 (33.7)	25,332 (34.7)	30,376 (32.5)
Missing	1032 (1.5)	1113 (1.8)	1097 (1.5)	1655 (1.8)
Smoking status				
Non-smoker	14,499 (21.4)	15,163 (24.8)	15,167 (20.8)	23,020 (24.6)
Current smoker	14,429 (21.3)	13,395 (21.9)	14,871 (20.4)	18,835 (20.2)
Ex-smoker	38,710 (57.2)	32,494 (53.2)	42,910 (58.8)	51,470 (55.1)
Missing	27 (0.0)	40 (0.1)	81 (0.1)	123 (0.1)
Ethnicity				
White	64,727 (95.7)	58,579 (95.9)	69,641 (95.4)	89,348 (95.6)
South Asian	1395 (2.1)	1002 (1.6)	1597 (2.2)	1399 (1.5)
Black	741 (1.1)	658 (1.1)	836 (1.1)	1015 (1.1)
Other	365 (0.5)	317 (0.5)	380 (0.5)	504 (0.5)
Mixed	189 (0.3)	159 (0.3)	223 (0.3)	253 (0.3)
Not stated	124 (0.2)	222 (0.4)	202 (0.3)	536 (0.6)
Missing	124 (0.2)	155 (0.3)	150 (0.2)	393 (0.4)
Index of Multiple Deprivation				
1 (least deprived)	15,473 (22.9)	15,030 (24.6)	16,682 (22.8)	23,770 (25.4)
2	15,130 (22.4)	13,989 (22.9)	16,437 (22.5)	21,641 (23.2)
3	13,527 (20.0)	12,142 (19.9)	14,538 (19.9)	18,436 (19.7)
4	12,205 (18.0)	10,682 (17.5)	13,227 (18.1)	15,999 (17.1)
5 (most deprived)	11,330 (16.7)	9249 (15.1)	12,145 (16.6)	13,602 (14.6)
Alcohol consumption				
Non-drinker	6196 (9.2)	5279 (8.6)	6836 (9.4)	7896 (8.4)
Current low level	30,430 (45.0)	28,857 (47.2)	30,651 (42.0)	42,126 (45.1)
Current Medium level	6480 (9.6)	6826 (11.2)	7234 (9.9)	11,153 (11.9)
Current high level	2640 (3.9)	2599 (4.3)	4028 (5.5)	4961 (5.3)
Ex-drinker	6198 (9.2)	5058 (8.3)	5807 (8.0)	6668 (7.1)
Missing	1884 (2.8)	1841 (3.0)	2187 (3.0)	3267 (3.5)
Systolic blood pressure*(in quartile)*^*a*^				
Q1	19,443 (28.7)	15,550 (25.5)	20,042 (27.4)	23,022 (24.6)
Q2	14,823 (21.9)	13,115 (21.5)	15,861 (21.7)	20,010 (21.4)
Q3	16,842 (24.9)	15,219 (24.9)	18,338 (25.1)	23,301 (24.9)
Q4	13,333 (19.7)	13,465 (22.0)	15,032 (20.6)	20,554 (22.0)
Missing	3224 (4.8)	3743 (6.1)	3756 (5.1)	6561 (7.0)
Diastolic blood pressure *(in quartiles)*^*b*^				
Q1	25,827 (38.2)	19,304 (31.6)	26,608 (36.4)	28,620 (30.6)
Q2	10,384 (15.3)	8727 (14.3)	11,186 (15.3)	13,731 (14.7)
Q3	14,856 (22.0)	13,985 (22.9)	16,012 (21.9)	20,905 (22.4)
Q4	13,330 (19.7)	15,286 (25.0)	15,413 (21.1)	23,577 (25.2)
Missing	3268 (4.8)	3790 (6.2)	3810 (5.2)	6615 (7.1)
Region^c^				
Northeast	2387 (3.5)	2155 (3.5)	2843 (3.9)	3432 (3.7)
Northwest	14,664 (21.7)	12,324 (20.2)	14,010 (19.2)	16,051 (17.2)
Yorkshire & The Humber	2716 (4.0)	2279 (3.7)	2850 (3.9)	3586 (3.8)
East Midlands	1712 (2.5)	1671 (2.7)	1595 (2.2)	1989 (2.1)
West Midlands	11,522 (17.0)	11,089 (18.2)	11,702 (16.0)	16,679 (17.8)
East of England	3484 (5.1)	3216 (5.3)	3484 (4.8)	5095 (5.5)
London	8664 (12.8)	7600 (12.4)	8248 (11.3)	9905 (10.6)
Southeast	14,487 (21.4)	13,559 (22.2)	16,602 (22.7)	21,371 (22.9)
Southwest	8026 (11.9)	7193 (11.8)	11,694 (16.0)	15,336 (16.4)
Missing	<5	6 (0.0)	<5	<5
Polypharmacy (≥5 drugs)	64,275 (95.0)	46,263 (75.7)	68,572 (93.9)	66,331 (71.0)
*Medical history*				
Any bleeding	38,732 (57.2)	26,417 (43.2)	42,413 (58.1)	44,659 (47.8)
Chronic kidney disease				
Stage 3a	25,827 (38.2)	19,304 (31.6)	26,608 (36.4)	28,620 (30.6)
Stage 3b	10,384 (15.3)	8727 (14.3)	11,186 (15.3)	13,731 (14.7)
Stage 4	14,856 (22.0)	13,985 (22.9)	16,012 (21.9)	20,905 (22.4)
Stage 5	13,330 (19.7)	15,286 (25.0)	15,413 (21.1)	23,577 (25.2)
Missing	3268 (4.8)	3790 (6.2)	3810 (5.2)	6615 (7.1)
COPD	12,940 (19.1)	7865 (12.9)	14,929 (20.4)	12,706 (13.6)
Diabetes	18,574 (27.4)	13,289 (21.8)	21,115 (28.9)	21,491 (23.0)
Heart failure	22,333 (33.0)	13,176 (21.6)	20,698 (28.3)	18,373 (19.7)
Ischaemic heart disease	33,685 (49.8)	19,708 (32.3)	32,534 (44.5)	25,977 (27.8)
Peripheral arterial disease	6404 (9.5)	3742 (6.1)	6197 (8.5)	4921 (5.3)
Peptic ulcer	8769 (13.0)	3380 (5.5)	10,311 (14.1)	4974 (5.3)
Stroke/TIA	12,318 (18.2)	8848 (14.5)	14,067 (19.3)	13,712 (14.7)
Venous thromboembolism	7349 (10.9)	4264 (7.0)	5523 (7.6)	5100 (5.5)
Medication use in the past 3 months				
ACEI/ARBs	41,310 (61.1)	32,111 (52.6)	39,612 (54.3)	44,170 (47.3)
Anticonvulsant	437 (0.6)	346 (0.6)	551 (0.8)	507 (0.5)
Antidepressant	6718 (9.9)	3682 (6.0)	8714 (11.9)	6449 (6.9)
Antiplatelet	33,655 (11.4)	7383 (10.9)	5982 (9.8)	11,149 (15.3)
Aspirin	98,392 (33.3)	22,941 (33.9)	26,633 (43.6)	23,475 (32.1)
Beta-blockers	43,356 (64.1)	37,136 (60.8)	48,513 (66.4)	58,441 (62.5)
CCBs	21,844 (32.3)	20,176 (33.0)	24,415 (33.4)	29,838 (31.9)
Macrolides	3373 (5.0)	2389 (3.9)	3330 (4.6)	3143 (3.4)
NSAIDs	10,380 (15.3)	5765 (9.4)	12,676 (17.4)	8793 (9.4)
Oestrogen/oestrogen like drugs	374 (0.6)	336 (0.5)	520 (0.7)	556 (0.6)
Oral corticosteroids	9455 (14.0)	3886 (6.4)	9743 (13.3)	6178 (6.6)
Statins	40,506 (59.9)	29,828 (48.8)	43,414 (59.4)	44,050 (47.1)
No. of GP active consultation in the past year				
Median (IQR)	19 (12–29)	13 (8–21)	15 (9–23)	11 (7–18)
Min, max	0, 199	0, 297	0, 221	0, 311
≥12 visits	51,838 (76.6)	35,736 (58.5)	48,102 (65.9)	45,673 (48.9)
<12 visits	15,476 (22.9)	24,671 (40.4)	24,725 (33.9)	47,086 (50.4)
None	351 (0.5)	685 (1.1)	202 (0.3)	689 (0.7)

*DOAC*, direct oral anticoagulants, *PPIs* proton pump inhibitors, *IQR* interquartile range, *COPD* chronic obstructive pulmonary disease, *TIA* transient ischaemic attack, *ACEI/ARBs* angiotensin-converting enzyme inhibitors/angiotensin receptor blockers, *CCBs* calcium channel blockers, *NSAIDs* non-steroidal anti-inflammatory drugs, *GP* general practice.

^a^Systolic blood pressure in quartile: Warfarin cohort: Q1 (40–120), Q2 (120.4–130), Q3 (131–140), Q4 (140.5–238); DOAC cohort: Q1 (40–120), Q2 (121–130), Q3 (130.2–140), Q4 (141–239).

^b^Diastolic blood pressure in quartile: Warfarin cohort: Q1 (30–70), Q2 (71–76), Q3 (77–80), Q4 (81–183); DOAC cohort: Q1 (30–70), Q2 (71–76), Q3 (77–81), Q4 (82–174).

^c^Round to nearest 5 due to data redaction.

**Table 2:** Baseline characteristics in CDARS before propensity score weighting

	Warfarin + PPI	Warfarin only	DOAC + PPI	DOAC only
Total	8491	8364	14,714	16,648
Age at index date				
Median (IQR)	74.6 (64.7–82.1)	72.1 (62.9–80.1)	78.9 (70.4–84.8)	76.2 (67.7–82.8)
Min, max	18.9, 101.3	21.0, 101.6	26.8, 109.8	18.6, 104.4
Age group				
18– < 40	75 (0.9)	125 (1.5)	38 (0.3)	72 (0.4)
40– < 50	271 (3.2)	303 (3.6)	162 (1.1)	264 (1.6)
50– < 60	919 (10.8)	1101 (13.2)	802 (5.5)	1186 (7.1)
60– < 70	1987 (23.4)	2202 (26.3)	2554 (17.4)	3733 (22.4)
70– < 80	2485 (29.3)	2504 (29.9)	4482 (30.5)	5458 (32.8)
80+	2754 (32.4)	2129 (25.5)	6676 (45.4)	5935 (35.6)
Male sex	4546 (53.5)	4442 (53.1)	7248 (49.3)	8348 (50.1)
Calendar year at cohort entry				
2011	610 (7.2)	1139 (13.6)	126 (0.9)	364 (2.2)
2012	673 (7.9)	1158 (13.8)	261 (1.8)	686 (4.1)
2013	813 (9.6)	1116 (13.3)	490 (3.3)	975 (5.9)
2014	999 (11.8)	1108 (13.2)	818 (5.6)	1259 (7.6)
2015	1148 (13.5)	1000 (12.0)	1361 (9.2)	1671 (10.0)
2016	1188 (14.0)	858 (10.3)	1821 (12.4)	2091 (12.6)
2017	1133 (13.3)	752 (9.0)	2549 (17.3)	2580 (15.5)
2018	1073 (12.6)	694 (8.3)	3036 (20.6)	3031 (18.2)
2019	854 (10.1)	539 (6.4)	4252 (28.9)	3991 (24.0)
Polypharmacy (≥5 drugs)	8134 (95.8)	6696 (80.1)	13,743 (93.4)	13,114 (78.8)
Medical history				
Alcohol-related liver disease	234 (2.8)	131 (1.6)	280 (1.9)	231 (1.4)
Any bleeding	2158 (25.4)	1128 (13.5)	3109 (21.1)	2200 (13.2)
Chronic kidney disease	803 (9.5)	346 (4.1)	365 (2.5)	233 (1.4)
COPD	787 (9.3)	523 (6.3)	1286 (8.7)	948 (5.7)
Diabetes	2213 (26.1)	1451 (17.3)	3170 (21.5)	2831 (17.0)
Heart failure	3043 (35.8)	1599 (19.1)	3242 (22.0)	2236 (13.4)
Hypertension	4137 (48.7)	3117 (37.3)	7299 (49.6)	6800 (40.8)
Ischaemic heart disease	2493 (29.4)	1149 (13.7)	3863 (26.3)	1976 (11.9)
Peripheral arterial disease	166 (2.0)	58 (0.7)	156 (1.1)	86 (0.5)
Peptic ulcer	1216 (14.3)	548 (6.6)	1879 (12.8)	971 (5.8)
Stroke/TIA	1168 (13.8)	838 (10.0)	1963 (13.3)	1648 (9.9)
Venous thromboembolism	618 (7.3)	331 (4.0)	266 (1.8)	130 (0.8)
Medication use in the past 3 months				
ACEI/ARBs	4507 (53.1)	3682 (44.0)	7594 (51.6)	7369 (44.3)
Anticonvulsant	1040 (12.2)	411 (4.9)	1531 (10.4)	867 (5.2)
Antidepressant	501 (5.9)	336 (4.0)	963 (6.5)	742 (4.5)
Antiplatelet	1361 (16.0)	482 (5.8)	2513 (17.1)	788 (4.7)
Aspirin	4712 (55.5)	5153 (61.6)	8579 (58.3)	9114 (54.7)
Beta-blockers	5146 (60.6)	4801 (57.4)	9050 (61.5)	10,104 (60.7)
CCBs	4274 (50.3)	4443 (53.1)	8055 (54.7)	9734 (58.5)
Macrolides	423 (5.0)	250 (3.0)	494 (3.4)	315 (1.9)
NSAIDs	779 (9.2)	434 (5.2)	1299 (8.8)	790 (4.7)
Oestrogen/oestrogen like drugs	10 (0.1)	9 (0.1)	21 (0.1)	20 (0.1)
Oral corticosteroids	1110 (13.1)	558 (6.7)	1663 (11.3)	957 (5.7)
Statins	4566 (53.8)	3527 (42.2)	9305 (63.2)	8498 (51.0)

*DOAC* direct oral anticoagulants, *PPIs* proton pump inhibitors, *IQR* interquartile range, *COPD* chronic obstructive pulmonary disease, *TIA* transient ischaemic attack, *ACEI/ARBs* angiotensin-converting enzyme inhibitors/angiotensin receptor blockers, *CCBs* calcium channel blockers, *NSAIDs* non-steroidal anti-inflammatory drugs.

In CPRD, ≥95% of the study population was of white ethnicity. In general, most ***OAC*** + ***PPI*** users were more likely to be older, live with obesity, have higher levels of deprivation, be former smokers, have higher levels of current alcohol consumption, have more co-morbidities (except VTE), have more co-medications (except aspirin and calcium channel blockers [CCBs]) and polypharmacy than ***OAC only*** groups. They also tended to have more general practice active consultation in the past year (Table 1).

In CDARS, ***OAC*** + ***PPI*** users were more likely to be older, have more co-morbidities, and have more co-medications (except CCBs) and polypharmacy than ***OAC only*** groups (Table 2).

In case-crossover studies, we identified 44,530 and 7155 GIB who had valid follow-up during the study period in CPRD and CDARS, respectively.

### Comparison between **warfarin**+**PPI** and **warfarin only**

**Fig. 1:**
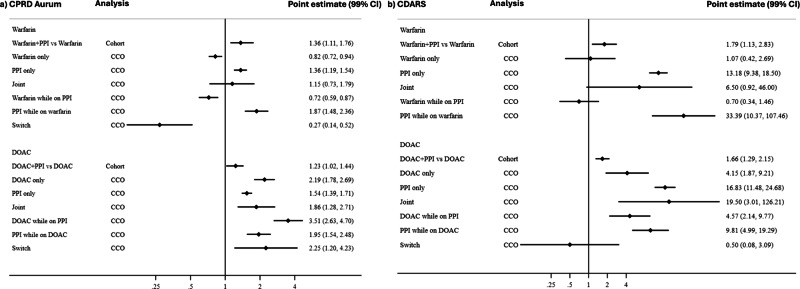
Results of the association between concomitant use of proton pump inhibitors and oral anticoagulants and gastrointestinal bleeding in cohort and case-crossover studies. CPRD Clinical Practice Research Datalink, CDARS Clinical Data Analysis and Reporting System, CCO case-crossover, CI confidence interval, DOAC direct anticoagulant, PPI proton pump inhibitors. Note: The parameter without an eligible estimate was not shown in the figure which is because of an extremely small number of events. Point estimate refers to the hazard ratio for cohort study analysis and the odds ratio for case-crossover study analysis.

In cohort design, higher hazard of GIB was found in ***warfarin*** + ***PPI***, compared to ***warfarin only*** in both settings (CPRD: HR = 1.36, 99% CI = 1.11–1.76; CDARS: HR = 1.79 99% CI = 1.13–2.83)) (Fig. 1, Supplementary Table 2).

In case-crossover design, the OR for GIB associated with ***warfarin only*** was 0.82 (99% CI = 0.72–0.94), and the OR for GIB associated with ***warfarin while on PPI*** was 0.72 (99% CI = 0.59–0.87). Higher ORs for GIB were similarly associated with ***PPI only*** (1.36, 99% CI = 1.19–1.54) and ***PPI while on warfarin*** (1.87, 99% CI = 1.48–2.36) in CPRD. In CDARS, no difference was seen in odds for GIB associated with warfarin only (1.07; 99% CI = 0.42–2.69). Higher ORs for GIB were associated with ***PPI only*** (13.18, 99% CI = 9.38–18.50) and ***PPI while on warfarin*** (33.39, 99% CI = 10.37–107.46) (Fig. 1). Wide CIs were observed for other parameters.

### Comparison between **DOAC**+**PPI** and **DOAC only**

In cohort design, a higher hazard of GIB was found in ***DOAC*** + ***PPI***, versus ***DOAC only*** in both settings (CPRD: HR = 1.23, 99% CI = 1.02–1.44; CDARS: HR = 1.66, 99% CI = 1.29–2.15)) (Fig. 1, Supplementary Table 3).

In case-crossover design, higher ORs for GIB were observed in all relevant parameters with comparable ORs (***DOAC only***: 2.19, 99% CI = 1.78–2.69; ***PPI only***: 1.54, 99% CI = 1.39–1.71; ***Joint***: 1.86, 99% CI = 1.28–2.71, ***DOAC while on PPI***: 3.51, 99% CI = 2.63–4.70, ***PPI while on DOAC***: 1.95, 99% CI = 1.54–2.48) in CPRD. In CDARS, higher ORs for GIB were associated with ***DOAC only*** (4.15, 99% CI = 1.87–9.21), ***Joint*** (19.50, 99% CI = 3.01–126.21) and ***PPI only*** (16.83, 99% CI = 11.48–24.68) (Fig. 1).

### Subgroup analysis

Stratified analyses by DOAC dose and DOAC type found no evidence of effect modification in cohort study design in both settings (Supplementary Tables 4–7). While there was no evidence that history of GIB was an effect modifier in CPRD, by contrast, in CDARS, we found an increased hazard of GIB only in people without a history of GIB for both ***warfarin*** + ***PPI*** (HR = 4.19 99% CI = 2.44–7.18) and ***DOAC*** + ***PPI*** (HR = 3.69, 99% CI = 2.57–5.31), interaction *p* < 0.01 (Supplementary Table 8).

### Sensitivity analysis

In cohort design, all sensitivity analyses showed similar results to the main analysis.

In case-crossover design, the ORs for GIB associated with ***warfarin only*** decreased by varying the length of hazard period from 7 to 90 days. We also observed the ORs for ***PPI only*** and ***PPI while on warfarin*** increased with increased length of hazard period in CPRD but not in CDARS (Fig. S3). Inadequate power was obtained in CDARS (Supplementary Fig. 1).

In CPRD, similar ORs were observed for all parameters in 7-day. However, the OR associated with ***PPI while on DOAC*** (2.13; 99% CI = 1.68–2.71) was lower than ***DOAC only*** (5.36; 99% CI = 4.06–7.08) with non-overlapped CIs in 90-day. Similarly, inadequate power was obtained in CDARS (Supplementary Fig. 2). In CDARS, the OR associated with ***PPI while on DOAC*** (10.57; 99% CI = 5.14–21.72) was higher than ***DOAC only*** (4.45; 99% CI = 1.89–10.52) with overlapped CIs in 90-day.

### Quantitative bias analysis

To potentially fully explain the PS-HR, an unmeasured confounder would need to be associated (conditional on measured covariates) with either ***warfarin*** + ***PPI/DOAC*** + ***PPI*** or GIB with a risk ratio of at least 2.06/1.76 (effect estimate) or 1.46/1.16 (lower bound) in CPRD Aurum and 2.98/2.71 (effect estimate) or 1.51/1.90 (lower bound) in CDARS respectively (Supplementary Fig. 3).

## Discussion

Amongst ~350,000 OAC users with AF in England and Hong Kong, higher hazards of GIB were found in ***OAC*** + ***PPI*** users, versus ***OAC only*** users but the evidence was modest. The case-crossover design showed higher odds for GIB in most concomitant PPI and OAC parameters and their estimates were comparable to ***PPI only*** or ***OAC only***, suggesting no evidence that PPIs modified the risk of GIB in OAC users.

Although we observed an increased risk of GIB in both settings using cohort design, we cannot exclude the possibility of confounding by indication. We found that ***PPI*** + ***OAC*** users had more co-morbidities and co-prescription than ***OAC only*** users, regardless of type of OAC. Using propensity score (PS) weighting, the estimates largely shifted towards null, and the SMDs were low, suggesting a considerable impact of confounding in our analysis. Similar to other observational studies using data from population-based databases, we do not have complete data on frailty or severity of the disease when PPI was commenced in OAC users. Our quantitative bias analysis showed that moderate strength of a missing confounder, for example, frailty, can potentially explain the findings. Furthermore, in our subgroup analysis, the hazard of GIB does not increase with the level of DOAC dose, further supporting that unmeasured confounding rather than combined use of PPI and DOAC explained the observed risk of GIB.

Importantly, we also conducted case-crossover design to support the interpretation of our findings by assessing and detecting potential time-varying confounder. First, the OR associated with ***PPI while on warfarin*** was not lower than ***warfarin only***, hence suggesting no evidence of protective effect of PPI against hospitalised GIB associated with warfarin. Surprisingly, we observed a reduced risk with GIB for ***warfarin only*** in 30 and 90 days which could be due to the decreasing prescribing trend of warfarin in England^15^. This would affect the assumption of case-crossover design that there is no population trend of prescribing over time. Therefore, the case-crossover design could be more susceptible to bias when the length of hazard period increases. However, we did not observe this pattern in Hong Kong (where we noticed only a small reduction from ~4% to ~2% incident warfarin users each year in all new OAC users during the study period). A more abrupt reduction in warfarin use was seen in England with a change from ~8% to ~1% in our cohorts. This demonstrates that using multiple datasets could help interpretation of the findings. For DOAC, increased odds in all parameters with comparable OR were found in both settings, suggesting no protective effect of PPI against hospitalised GIB in DOAC users. In our case-crossover sensitivity analysis, although we observed a lower OR of GIB associated with initiating ***PPI while on DOAC*** compared with initiating ***DOAC only*** in 90-day in CPRD with non-overlapped CIs, similar pattern was not found in 7-day or 30-day nor any length of hazard window in CDARS. Therefore, the evidence of protective effect of PPI against hospitalised GIB in DOAC users was very weak.

Four studies showed a lower risk of upper GIB associated with co-prescribed PPIs and OACs for one year^3,4,16^ and one study showed that the protective effect was only observed in rivaroxaban and warfarin users with a history of GIB^5^. A randomised controlled trial (RCT) showed a 50% reduced risk of overt bleeding of gastroduodenal origin comparing pantoprazole with placebo^17^. However, no difference in risk of upper GIB (composite of overt bleeding of gastroduodenal origin and unknown origin, bleeding of presumed occult upper GI tract origin) between pantoprazole and placebo, but the trial size had limited power to detect rarer outcomes (*n* = 17,598)^17^. Regarding absolute risks for studies comparing risk of GIB among ***warfarin*** + ***PPI*** users versus ***warfarin only*** users, absolute risks of GIB varied from 0.36–6.53% in different populations reported from the UK, Hong Kong, and United States^4^. The highest absolute risk of GIB in Hong Kong was 6.53% in people prescribed warfarin only. Notably, the outcome of interest in the US study was upper GIB only, and the population was not limited to AF patients only^4^, which was not comparable to our study. For studies comparing risk of GIB among ***DOAC*** + ***PPI*** users versus ***DOAC only*** users, absolute risks of GIB were similar and ranged from 0.33–0.77% in the UK and US^4^. However, edoxaban was not included in the US study. The absolute risk of GIB in Hong Kong was similar to that of the RCT^17^. However, this RCT only considered rivaroxaban. Their study population was stable coronary or peripheral arterial disease patients, and it ended halfway. Lee et al. (2022) combined both warfarin and DOAC as ***OAC with co-therapy of PPI*** compared with ***OAC only*** in people with AF in South Korea and the absolute risk of GIB was approximately 2%^5^. Another cohort study also showed no difference in risk of GIB between using gastroprotective agent users and non-users among people with DOACs, but the cohort size was small (*n* = 2076) without absolute risk reporting^18^.

To date, this is the first population-based study using both datasets from England and Hong Kong investigating the effects of PPIs on GIB in OAC users. Second, this study utilised two study designs and robust methods to capture signals, detect and reduce confounding. Our study population mostly consists of White and Chinese; results are therefore generalisable to these ethnic groups.

This study has some limitations. First, drug adherence was unknown, and over-the-counter PPI was not captured in both databases, leading to potential misclassification bias of exposure. However, assuming a non-differential misclassification of exposure, estimates would only be biased towards null. Additionally, we included hospitalised GIB as outcome of interest to ensure we captured incident cases. However, this means our cases are likely to represent severe cases only. Moreover, although the proportion of missingness was very low in our study, the assumption of ‘missing at random’ may not hold for all covariates with missingness in CPRD. Lastly, we could not eliminate residual confounding (e.g., frailty and severity of disease), but we attempted to minimize confounding by using a PS method and assessed the impact of potential residual confounding on our analyses using quantitative bias analysis. Importantly, we performed the 6-parameter model case-crossover design to reduce confounding and help assess and detect potential time-varying confounding when the drugs were initiated.

Our study shows that there is no safety concern in co-prescribing PPIs and OACs, but we do not provide evidence to recommend the combination of PPIs and OACs. Further robust evidence on the role of PPIs in reducing GIB among OAC users is needed to inform further clinical recommendations. In particular, future studies of larger sample sizes could focus on users of individual DOACs, specifically apixaban, and edoxaban, which have been increasingly prescribed compared with other DOACs.

Overall, the elevated risk of GIB associated with the combined use of OAC and PPIs we observed could be largely explained by residual confounding. We did not find ethnic variations in the effect of GIB associated with PPIs in OAC users, but using different population-based datasets could help interpretation. Using novel case-crossover design can also help detect and assess residual confounding in studies investigating the impact of concomitant use of drugs.

## Methods

### Study design

**Fig. 2:**
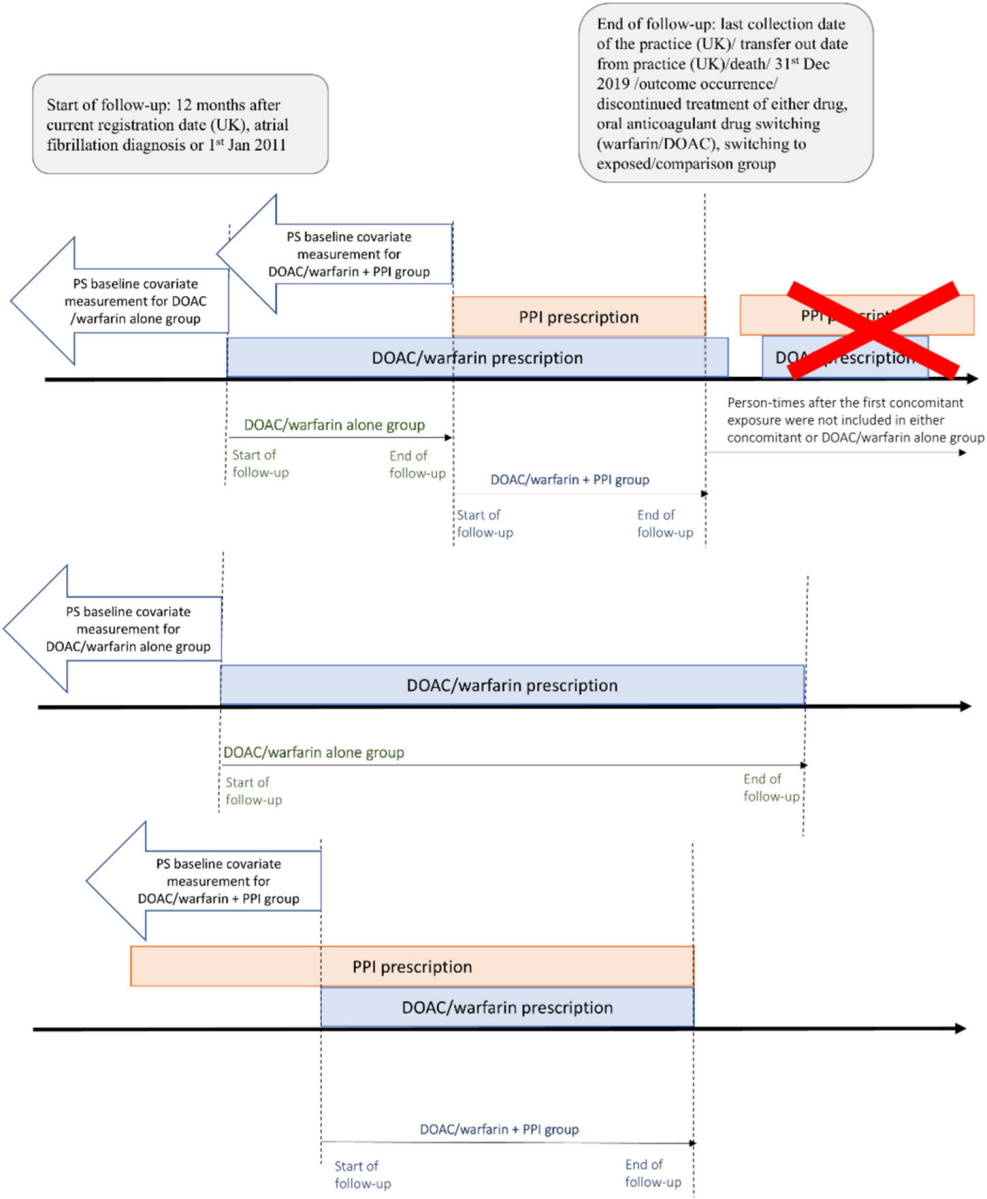
Examples of inclusion of study cohorts in cohort study design. PS propensity score, UK United Kingdom, DOAC direct oral anticoagulant, PPI proton pump inhibitors.

**Fig. 3:**
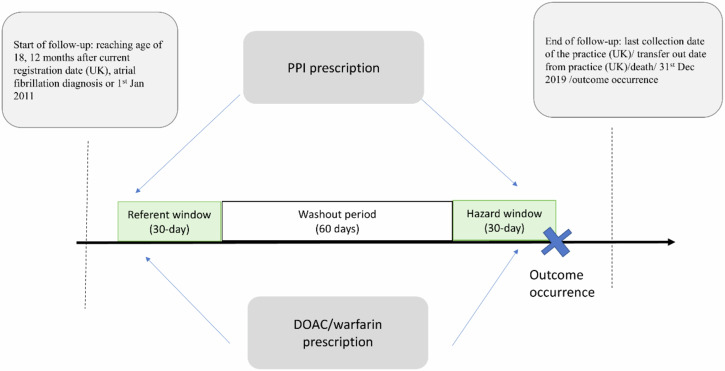
Illustration of the modified case-crossover design. UK United Kingdom, DOAC direct oral anticoagulant, PPI proton pump inhibitors.

We used both cohort and case-crossover study design as an optimal combination of study design which could provide absolute risks for quantifying drug-drug interactions and eliminate between-person confounding respectively (Figs. 2–3, Tables 3–4).

**Table 3:** Case-crossover study design 6-parameter model

Case-crossover study design 6-parameter model
1. Initiation of OAC only (***DOAC/warfarin only***)
2. Initiation of PPI only (***PPI only***)
3. Both OAC and PPI initiated together (***Joint***)
4. Initiation of OAC in the presence of PPI (***DOAC/warfarin while on PPI***)
5. Initiation of PPI in the presence of OAC (***PPI while on DOAC/warfarin***)
6. Use one drug (OAC/PPI) in the hazard window and the other drug (PPI/OAC) in the referent window (***Switch***)

*DOAC* direct oral anticoagulant, *PPI* proton pump inhibitors, *OAC* oral anticoagulant.

The parameter of ***Switch*** does not directly inform an assessment of the impact of concomitant use of drugs and is also unlikely of clinical interest, but it is included as it represents a possible exposure pattern parameter in the analysis of two concomitant uses of drugs. Notably, if we observe a relatively lower odds ratio associated with ***PPI while on OAC*** than ***OAC only*** with non-overlapped confidence intervals, it suggests that initiation of PPI in OAC users could reduce the risk of gastrointestinal bleeding associated with OACs. Using ***OAC while on PPI*** parameter, we are also able to assess whether the odds ratio for gastrointestinal bleeding associated with OACs in PPI users would be lower than ***OAC only***. The ***PPI only*** parameter can help detect time-varying confounding for other parameters by enabling comparisons. An observed increase in the odds of gastrointestinal bleeding in ***PPI only*** parameter could largely be explained by the underlying gastrointestinal condition requiring PPI use at the specific time point, rather than PPI itself. Similarly, for ***Joint*** parameter, multiple medical condition would require separate treatment present at that time point, therefore multimorbidity rather than the drugs could lead to increased risk of gastrointestinal bleeding. We illustrated the 6-parameter model in Table 4.

**Table 4:** Illustration of drug initiation patterns in case-crossover study design using 6-parameter model

Strata	Strata description	Hazard window		Referent window		Odds ratio (99% CI)
		DOAC/warfarin	PPI	DOAC/warfarin	PPI	
1	DOAC/warfarin only	1	0	0	0	
		0	0	1	0	Reference
2	PPI only	0	1	0	0	
		0	0	0	1	Reference
3	Joint exposure	1	1	0	0	
		0	0	1	1	Reference
4	DOAC/warfarin while on PPI	1	1	0	1	
		0	1	1	1	Reference
5	PPI while on DOAC/warfarin	1	1	1	0	
		1	0	1	1	Reference
6	Switch	1	0	0	1	
		0	1	1	0	Reference

*DOAC* direct oral anticoagulant, *PPI* proton pump inhibitors, *CI* confidence interval.

### Assumptions for cohort study design and case-crossover study design

To investigate possible causal associations in studies investigating the effect of concomitant drug exposure, conventional observational study designs, including cohort studies, could be used. However, cohort design is vulnerable to between-person confounding because the risk of the outcomes of interest is compared between those receiving concomitant drug exposure and the comparison group (those not receiving concomitant drugs. As a within-person study design, the case-crossover study can eliminate time-invariant confounding but cannot estimate absolute risk. On the other hand, a cohort design could estimate absolute risks which can then be used to examine public health impact. Therefore, cohort study and case-crossover study designs are an optimal combination to obtain both robust relative and absolute measures of effect.

### Data source

We used data from the Clinical Practice Research Datalink (CPRD) Aurum, which contains primary care records of more than 13 million currently registered patients from 1491 general practices in the UK using EMIS software systems^19,20^. We also used linked death data from the Office for National Statistics (ONS), hospital admissions data from Hospital Episode Statistics Admitted Patient Care (HES APC), and deprivation data from the Index of Multiple Deprivation^19,20^.

In Hong Kong, ~7.6 million citizens have access to the public healthcare setting which provides >90% of inpatient services^21^. The Clinical Data Analysis and Reporting System (CDARS), managed by Hong Kong Hospital Authority (HA) contains all diagnostic and prescription records in public clinics and hospitals.

## Cohort study

### Exposure and comparator

We identified people with AF aged ≥18 years receiving their first warfarin or DOAC (dabigatran, rivaroxaban, apixaban and edoxaban), with acceptable research quality during the study period (1/1/2011-31/12/2019). To ensure that we have reliable measures of drug use and baseline covariates, all participants had ≥1 year continuous registration before the first recorded DOAC prescription in CPRD. This criterion is not needed for CDARS as all Hong Kong residents have access to public healthcare services since birth, so all their public healthcare records were available since 1993^21^.

The exposure groups were defined as overlapping time when OAC and PPI were prescribed concurrently. The overlapping patterns could be 1) both OAC and PPI were prescribed on the same day, or 2) one drug was prescribed first and another drug was then prescribed concurrently. We performed two separate analyses by comparing: 1) people who had concurrent prescriptions of warfarin and a PPI (***warfarin*** + ***PPI***) with those who had warfarin prescription only (***warfarin only***, comparator), and 2) those who had concurrent prescriptions of a DOAC with a PPI (***DOAC*** + ***PPI***) with those who had a DOAC prescription only (***DOAC only***, comparator). People with another type of OAC prescription before cohort entry were excluded to remove a carry-over effect. The duration of prescriptions for OACs and PPIs was calculated and used to determine the comparison groups. We estimated the prescription duration according to the recorded duration, or by calculation using quantity, dosage and frequency. If there was any missingness or extreme values (based on the recommended treatment duration, or a maximum of three months for a prescription refill for ongoing therapies), we imputed the duration with the study population median for that drug. We assumed the treatment discontinued when there was a treatment gap of >1 day between each prescription to lower the misclassification bias of exposure. The exposure groups were defined as person-time when an OAC and PPI or OAC were prescribed concurrently (Fig. 2).

### Outcome

We identified the first hospitalised record of GIB during the follow-up to capture incident events. Previous studies showed high accuracy to capture GIB using HES (~90%) and CDARS (≥90%), respectively^3,22^. We followed all groups from index date (date of overlapped DOAC/warfarin and PPI prescription for the exposed group and date of first of DOAC/warfarin prescription for the OAC only group) until the earliest of discontinued treatment of either drug (OAC/PPI), switching to either group, GIB occurrence, death, transfer out of the practice (CPRD only, assuming no migration away from Hong Kong), last data collection date for the practice (CPRD only) or end of the study (31/12/2019).

### Covariates

The following covariates that are potential confounders were accounting for in the analyses (except if the variable was the exposure/outcome of interest): lifestyle factors including smoking status (chronic obstructive pulmonary disease as proxy in CDARS) and alcohol consumption (alcohol-related liver disease as proxy in CDARS), body mass index (CPRD Aurum only), deprivation status (CPRD Aurum only), region (CPRD Aurum only), age, sex, co-morbidities (chronic obstructive pulmonary disease, chronic renal disease, heart failure, ischaemic heart disease, ischaemic stroke/transient ischaemic attack, diabetes mellitus, peripheral arterial disease, VTE, bleeding, peptic ulcer), measurements of systolic and diastolic blood pressure in the past year (hypertension diagnosis as proxy in CDARS), prescriptions used in the past 3 months (aspirin, antiplatelets, antidepressants, anticonvulsants, angiotensin-converting enzyme inhibitors, non-steroidal anti-inflammatory drugs, oral corticosteroids, macrolides), polypharmacy, number of GP active consultation in the past year (CPRD Aurum only). Smoking status, body mass index, and alcohol consumption were pragmatically based on status recorded closest to the first day of follow-up. Records within −1 year to +1 month from the first day of follow-up were regarded as the best, +1 month to +1 year from the first day of follow-up being second best, the nearest before −1 year from the first day of follow-up as the third best, and within +1 year from the first day of follow-up being least good.

### Statistical analyses

To reduce confounding, PS was used to re-weight the sample and achieve a balance between groups on observed covariates. The PS is the probability of a patient receiving a certain treatment, based on the distribution of confounders among patients^23^. We derived PS from logistic regression, to represent the probability of exposure given the covariates measured on the first day of follow-up in each group. Weights are calculated as the inverse of PS for the exposed group and the inverse of (1-PS) for the comparison group. The balance of covariate distribution was assessed after weighting by calculating the standardized difference for each covariate. It is noted that, in the CDARS setting, we initially pre-specified 95% confidence intervals for all analyses. To deal with multiple tests and to parallel the analyses in the CPRD setting, we decided to present all results in 99% confidence intervals (CIs) before running all analyses. We finally computed hazard ratios (HRs) of the association and robust standard errors using inverse probability of treatment-weighted Cox regressions with 99% CI to handle multiple testing. We also tested for a non-zero slope of the scaled Schoenfeld residuals to explore the proportional hazards. There was no evidence of assumption violation for primary analysis in CPRD but not in CDARS. We therefore conducted further sensitivity analysis ending the follow-up up to 7 days since cohort entry and observed no evidence of assumption violation with the follow-up of 7-day. Similar results to the primary analysis were found but with wider CIs (warfarin: PS-HR 1.40, 99% CI 0.28–6.94; DOAC: PS-HR 1.44, 99% CI 0.73–2.88).

Multiple imputation through chained equations, including exposure, outcome, and PS covariates variables in the model with ten imputed datasets, was used to address the covariate missingness in the PS method, assuming data were missing at random. We estimated the treatment effect from each imputed dataset, followed by combining the treatment effect estimates for an overall estimate using Rubin’s rules^24^. We restricted the cohort to those individuals whose PS were within the overlapping region of the distributions of the exposed and comparison groups to reduce potential effects of residual confounding^23^.

### Subgroup and sensitivity analyses

To reduce confounding, PS was used to re-weight the sample and achieve a balance between groups on observed covariates. First, as some individuals could contribute person-times both in the concomitant group and OAC-only group, which might have led to overconfidence in our estimates, we computed the 99% confidence interval (CI) using bias-corrected bootstrapping method with 100 iterations. Analyses were stratified by history of GIB, level of DOAC dose (using the strength of DOAC as proxy), and individual DOAC. High dose of DOAC for AF was defined when drug strength was 150 mg for dabigatran, 20 mg for rivaroxaban, 5 mg for apixaban, and 60 mg for edoxaban, respectively, while low dose was defined as 110 mg for dabigatran, 15 mg for rivaroxaban, 2.5 mg for apixaban, and 30 mg for edoxaban^25–28^.

### Quantitative bias analysis

A post-hoc E-value was used to estimate the minimum necessary strengths of association between an unmeasured confounder and exposure or outcome, conditional on measured covariates, to potentially fully explain the observed non-null adjusted associations^29^.

## Modified case-crossover study

The case-crossover design eliminates time-invariant confounding as risks are compared within the individual^30^. It only includes individuals who experienced the outcome (cases) and compares each individual’s exposure in a time period prior to the outcome (hazard window) to the exposure during an earlier control period (referent window)^31^. This design has recently been developed and implemented to study effects of concomitant drug use with different drug initiation patterns, by performing 6-parameter model^31,32^.

In each case-crossover analysis, we identified people who experienced the first GIB and were exposed to PPI and/or OAC prior to GIB during a valid follow-up, which started from the latest of first AF diagnosis, study start date (1/1/2011) or ≥1 year continuous registration of General Practices (CPRD only), reaching age of 18 until outcome occurrence (i.e., index date), death, transfer out of the practice (CPRD only), last data collection date for the practice (CPRD only), or end of the study (31/12/2019). The hazard window started from days 1–30 on/before the diagnosis date of GIB. The referent window started from days 91–120 before the diagnosis date. We added a 60-day washout period to avoid auto-correlation in exposure between periods and carry-over effects. Drug exposure was defined based on at least one drug prescription during the hazard or referent window. Only discordant pairs of exposure status between hazard and referent windows contributed to the analyses (Fig. 3).

We used conditional logistic regression to compare the odds of exposure to the drugs during the hazard window to the odds of exposure in the referent window, conditioned on individuals with 99% CI. We estimated the odds ratios (ORs) for GIB associated with different drug initiation patterns using the 6-parameter model (Tables 3 and 4).

### Subgroup and sensitivity analyses

We stratified the analyses by doses and types of DOACs. As a sensitivity analysis, we repeated the analysis using 7-day and 90-day hazard and referent windows to investigate the sensitivity of results to the choice of risk period length.

Stata/MP 17, 18, and R 4.3.1 were used for all data processing and analyses.

## Supplementary information

Supplementary materials

## Data Availability

Computing code and study protocol are available from the corresponding author upon request for reproducing the results. However, the study data cannot be made available to other researchers because of the terms specified in Data Use Agreements.
