# Supplementary material for: The association between proton pump inhibitors and the risk of gastrointestinal bleeding in oral anticoagulants users
Source: NPJ Cardiovasc Health. 2025 Apr 12;2:11. doi: 10.1038/s44325-024-00037-3 (PMC11993357; doi:10.1038/s44325-024-00037-3)
Supplement: Supplementary file 1 — Supplementary materials [file 44325_2024_37_MOESM1_ESM.pdf]

Supplementary materials: Effect of proton pump inhibitors on risk of gastrointestinal bleeding associated with oral anticoagulants users

Table of Contents

Supplementary Table 1. Standardised mean difference in cohort study design 2

Supplementary Table 2. Details of results from univariable and propensity score models comparing warfarin + proton pump inhibitors with warfarin only in cohort study design5

Supplementary Table 3. Details of results from univariable and propensity score models comparing direct oral anticoagulants + proton pump inhibitors with direct oral anticoagulant only in cohort study design 5

Supplementary Table 4. Subgroup analysis by level of dose of DOAC for the association between concomitant use of DOAC and PPI and gastrointestinal bleeding, compared with DOAC only in cohort study 6

Supplementary Table 5. Subgroup analysis by individual DOAC for the association between concomitant use of OAC and PPI and gastrointestinal bleeding, compared with OAC only in cohort study 6

Supplementary Table 6. Subgroup analysis by level of dose of DOAC for the association concomitant use of DOAC and PPI and gastrointestinal bleeding in case-crossover study 7

Supplementary Table 7. Subgroup analysis by individual DOAC for the association concomitant use of OAC and PPI and gastrointestinal bleeding in case-crossover study .8

Supplementary Table 8. Subgroup analysis by history of gastrointestinal bleeding for the association between concomitant use of OAC and PPI and gastrointestinal bleeding, compared with OAC only in cohort study 10

Supplementary Figure 1. Sensitivity analysis varying the length of hazard period for the association concomitant use of warfarin and PPI and gastrointestinal bleeding in case-crossover study..11

Supplementary Figure 2. Sensitivity analysis varying the length of hazard period for the association concomitant use of DOAC and PPI and gastrointestinal bleeding in case-crossover study..12

Supplementary Figure 3. E-value for the lower 99% confidence interval and point estimate 13

Supplementary Table 1. Standardised mean difference in cohort study design

	UK CPRD		Hong Kong CDARS	
	DOAC + PPI vs DOAC only	warfarin + PPI vs warfarin only	DOAC + PPI vs DOAC only	warfarin + PPI vs warfarin only
**Age group**				
**18-<40**	1 (ref)	1 (ref)	1 (ref)	1 (ref)
**40-<50**	0.0287	0.0151	-0.0021	0.0093
**50-<60**	0.0147	0.0045	-0.0097	-0.0100
**60-<70**	0.0199	0.0176	-0.0011	-0.0091
**70-<80**	0.0196	0.0118	-0.0055	-0.0150
**80+**	0.0075	0.0325	-0.0025	-0.0017
**Male sex**	0.0005	0.0043	0.0009	-0.0160
**Body mass index**				
**Underweight**	1 (ref)	1 (ref)	NA	NA
**Normal**	0.0037	0.0088	NA	NA
**Overweight**	0.0019	0.0125	NA	NA
**Obese**	0.0033	0.0048	NA	NA
**Smoking status**				
**Non-smoker**	1 (ref)	1 (ref)	NA	NA
**Current smoker**	0.0011	0.0062	NA	NA
**Ex-smoker**	0.0030	0.0042	NA	NA
**Index of Multiple Deprivation**				
**1 (least deprived)**	1 (ref)	1 (ref)	NA	NA
**2**	0.0017	0.0037	NA	NA
**3**	0.0015	0.0024	NA	NA
**4**	0.0036	0.0007	NA	NA
**5 (most deprived)**	0.0028	0.0004	NA	NA
**Alcohol consumption**				
**Non-drinker**	1 (ref)	1 (ref)	NA	NA
**Current low level**	0.0094	0.0098	NA	NA
**Current Medium level**	0.0063	0.0013	NA	NA
**Current high level**	0.0036	0.0216	NA	NA
**Ex-drinker**	0.0035	0.0222	NA	NA
**Current drinker with missing data on consumption level**	0.0041	0.0039	NA	NA
**Systolic blood pressure *(in quartile)***				
**Q1 (50-120 mmHg)**	1 (ref)	1 (ref)	NA	NA

**Q2 (121-130 mmHg)**	0.0093	0.0119	NA	NA
**Q3 (130.2-140 mmHg)**	0.0119	0.0049	NA	NA
**Q4 (141-238 mmHg)**	0.0040	0.0052	NA	NA
**Diastolic blood pressure** ***(in quartiles)***				
**Q1 (30-70 mmHg)**	1 (ref)	1 (ref)	NA	NA
**Q2 (71-76 mmHg)**	0.0084	0.0041	NA	NA
**Q3 (77-82 mmHg)**	0.0091	0.0024	NA	NA
**Q4 (83-162 mmHg)**	0.0064	0.0037	NA	NA
**Region**				
**Northeast**	1 (ref)	1 (ref)	NA	NA
**Northwest**	0.0004	0.0141	NA	NA
**Yorkshire & The Humber**	0.0018	0.0103	NA	NA
**East Midlands**	0.0011	0.0006	NA	NA
**West Midlands**	0.0004	0.0069	NA	NA
**East of England**	0.0012	0.0048	NA	NA
**London**	0.0020	0.0028	NA	NA
**Southeast**	0.0039	0.0006	NA	NA
**Southwest**	0.0048	0.0062	NA	NA
**Polypharmacy (≥5 drugs)**	0.0019	0.0051	0.0035	0.0176
**Medical history**				
**Alcohol-related liver disease**	NA	NA	0.0083	0.0059
**Any bleeding**	0.0033	0.0321	0.0247	0.0464
**Chronic kidney disease**	NA	NA	0.0161	0.0337
Stage 3a	0.0023	0.0174	NA	NA
Stage 3b	0.0051	0.0132	NA	NA
Stage 4	0.0017	0.0020	NA	NA
Stage 5	0.0161	0.0085	NA	NA
**COPD**	0.0061	0.0278	0.0077	0.0143
**Diabetes**	NA	NA	0.0011	0.0128
without insulin	0.0014	0.0015	NA	NA
with insulin	0.0047	0.0044	NA	NA
**Heart failure**	0.0033	0.0034	0.0166	0.0286
**Hypertension**	NA	NA	0.0165	0.0232
**Ischaemic heart disease**	0.0078	0.0383	0.0189	0.0447
**Peptic ulcer**	0.0106	0.1001	0.0267	0.0570
**Peripheral arterial disease**	0.0032	0.0196	0.0019	-0.0019
**Stroke/TIA**	0.0036	0.0098	0.0155	0.0203
**Venous thromboembolism**	0.0010	0.0233	-0.0006	0.0549

**Medication use in the past 3 months**				
**ACEI**	0.0022	0.0204	0.0513	0.0532
**Anticonvulsants**	0.0007	0.0026	-0,0178	0.0150
**Antidepressants**	0.0038	0.0243	0.0029	0.0214
**Antiplatelets**	0.0124	0.0174	0.0411	0.0974
**Aspirin**	0.0056	0.0139	-0.0118	-0.0318
**Macrolides**	0.0058	0.0260	0.0131	-0.0024
**NSAIDs**	0.0020	0.0493	0.0029	0.0111
**Oral corticosteroids**	0.0040	0.0631	0.0182	0.0311
**No of GP active consultation in the past year**				
**≥12 visits**	1 (ref)	1 (ref)	NA	NA
**<12 visits**	0.0079	0.0333	NA	NA
**None**	0.0113	0.0026	NA	NA

Abbreviation: DOAC: direct oral anticoagulant, PPI: proton pump inhibitors, COPD: chronic obstructive pulmonary disease, TIA: transient ischaemic attack, ACEI: angiotensin-converting enzyme inhibitor, NSAIDs: nonsteroidal anti-inflammatory drugs, GP: general practice

Supplementary Table 2. Details of results from univariable and propensity score models comparing warfarin + proton pump inhibitors with warfarin only in cohort study design

	UK CPRD							Hong Kong CDARS						
	Number of events	Total person-year	Rate per 1,000	Univariable model		Propensity score model		Number of events	Total person-year	Rate per 1,000	Univariable model		Propensity score model	
				HR	99% CI	HR	99% CI				HR	99% CI	HR	99% CI
warfarin only	220	10,021	21.95	1.00 (ref)		1.00 (ref)		94	8,679	10.83	1.00 (ref)		1.00 (ref)	
warfarin + PPI	290	5,771	52.06	1.97	1.55 – 2.50	1.36	1.11 – 1.76	148	4,654	31.80	2.53	1.79 – 3.56	1.79	1.13 – 2.83

Abbreviation: UK: United Kingdom, CPRD: Clinical Practice Research Datalink, CDARS: Clinical Data Analysis and Reporting System, HR: hazard ratio, CI: confidence interval, PPI: proton pump inhibitors

Note: The number of people excluded due to non-overlapping region of PS distribution ranged from 29 to 35 (0.02-0.03%) in 10 imputed datasets in UK CPRD and was 3 (0.02%) in Hong Kong CDARS.

Supplementary Table 3. Details of results from univariable and propensity score models comparing direct oral anticoagulants + proton pump inhibitors with direct oral anticoagulant only in cohort study design

	UK CPRD							Hong Kong CDARS						
	Number of events	Total person-year	Rate per 1,000	Univariable model		Propensity score model		Number of events	Total person-year	Rate per 1,000	Univariable model		Propensity score model	
				HR	99% CI	HR	99% CI				HR	99% CI	HR	99% CI
DOAC only	546	18,589	29.37	1.00 (ref)		1.00 (ref)		225	17,097	13.16	1.00 (ref)		1.00 (ref)	
DOAC + PPI	499	8,939	55.83	1.72	1.46 – 2.02	1.23	1.02 – 1.44	305	10,353	29.46	2.06	1.64 – 2.58	1.66	1.29 – 2.15

Abbreviation: UK: United Kingdom, CPRD: Clinical Practice Research Datalink, CDARS: Clinical Data Analysis and Reporting System, HR: hazard ratio, CI: confidence interval, DOAC: direct oral anticoagulant, PPI: proton pump inhibitor

Note: The number of people excluded due to non-overlapping region of PS distribution ranged from 55 to 75 (0.03-0.05%) in 10 imputed datasets in UK CPRD and was 25 (0.87%) in Hong Kong CDARS.

Supplementary Table 4. Subgroup analysis by level of dose of DOAC for the association between concomitant use of DOAC and PPI and gastrointestinal bleeding, compared with DOAC only in cohort study

	UK CPRD				HK CDARS			
**Dose level**	**PS-HR**	**99% lower CI**	**99% Upper CI**	**Interaction p-value**	**PS-HR**	**99% lower CI**	**99% Upper CI**	**Interaction p-value**
**Low dose**	1.17	0.88	1.57	0.72	1.65	1.21	2.26	0.80
**High dose**	1.23	0.98	1.55		1.80	1.08	3.01	

Abbreviation: DOAC: direct oral anticoagulant, PPI: proton pump inhibitors, UK: United Kingdom, CPRD: Clinical Practice Research Datalink, HK: Hong Kong, CDARS: Clinical Data Analysis and Reporting System, PS-HR: propensity score model hazard ratio, CI: confidence interval

Supplementary Table 5. Subgroup analysis by individual DOAC for the association between concomitant use of OAC and PPI and gastrointestinal bleeding, compared with OAC only in cohort study

	UK CPRD				HK CDARS			
**Individual DOAC**	**PS-HR**	**99% lower CI**	**99% Upper CI**	**Interaction p-value**	**PS-HR**	**99% lower CI**	**99% Upper CI**	**Interaction p-value**
**Dabigatran**	1.28	0.64	2.55	0.78	**1.52**	**1.07**	**2.17**	0.27
**Rivaroxaban**	**1.32**	**1.01**	**1.73**		1.65	1.00	2.73	
**Apixaban**	1.14	0.88	1.49		**2.09**	**1.19**	**3.68**	
**Edoxaban**	1.19	0.56	2.53		3.21	0.16	64.33	

Abbreviation: DOAC: direct anticoagulant, UK: United Kingdom, CPRD: Clinical Practice Research Datalink, HK: Hong Kong, CDARS: Clinical Data Analysis and Reporting System, PS-HR: propensity score model hazard ratio, CI: confidence interval, PPI: proton pump inhibitors

Supplementary Table 6. Subgroup analysis by level of dose of DOAC for the association concomitant use of DOAC and PPI and gastrointestinal bleeding in case-crossover study

	UK CPRD			HK CDARS		
	Odds ratio	99% lower CI	99% Upper CI	Odds ratio	99% lower CI	99% Upper CI
**Low dose**						
DOAC only	2.30	1.75	3.03	4.36	1.84	10.32
PPI only	1.57	1.43	1.72	16.15	11.25	23.17
both drugs were initiated together	2.03	1.17	3.51	17.00	2.61	110.76
initiation of DOAC in the presence of PPI	3.09	2.16	4.42	2.70	1.38	5.30
initiation of PPI in the presence of DOAC	1.90	1.28	2.80	8.73	3.84	19.81
Use one drug in the hazard window and the other drug in the control window	2.38	1.02	5.59	0.25	0.03	1.92
**High dose**						
DOAC only	1.64	1.28	2.10	2.29	0.71	7.35
PPI only	1.54	1.40	1.70	14.65	10.53	20.38
both drugs were initiated together	1.71	1.08	2.72	NA*	NA*	NA*
initiation of DOAC in the presence of PPI	2.17	1.60	2.96	1.73	0.65	4.59
initiation of PPI in the presence of DOAC	2.09	1.53	2.86	12.00	2.55	56.42
Use one drug in the hazard window and the other drug in the control window	1.48	0.74	2.96	0.14	0.01	2.24

Abbreviation: DOAC: direct anticoagulant, UK: United Kingdom, CPRD: Clinical Practice Research Datalink, HK: Hong Kong, CDARS: Clinical Data Analysis and Reporting System, CI: confidence interval, PPI: proton pump inhibitors, CKD: chronic kidney disease, NA: not applicable
*No event or extreme small number of events on this parameter so no eligible estimate was shown.

Supplementary Table 7. Subgroup analysis by individual DOAC for the association concomitant use of OAC and PPI and gastrointestinal bleeding in case-crossover study

	UK CPRD			HK CDARS		
	**Odds ratio**	**99% lower CI**	**99% Upper CI**	**Odds ratio**	**99% lower CI**	**99% Upper CI**
**Dabigatran**						
DOAC only	1.85	1.06	3.23	3.62	1.30	10.14
PPI only	1.56	1.42	1.70	15.79	11.14	22.38
both drugs were initiated together	3.17	0.95	10.58	19.00	1.35	266.98
initiation of DOAC in the presence of PPI	1.43	0.71	2.89	1.75	0.78	3.92
initiation of PPI in the presence of DOAC	1.90	0.93	3.87	8.75	3.35	22.88
Use one drug in the hazard window and the other drug in the control window	0.40	0.05	3.45	0.40	0.05	3.45
**Rivaroxaban**						
DOAC only	2.10	1.57	2.81	2.75	0.61	12.37
PPI only	1.57	1.43	1.72	15.03	10.75	21.02
both drugs were initiated together	1.89	1.10	3.23	NA*	NA*	NA*
initiation of DOAC in the presence of PPI	2.86	1.94	4.23	2.37	0.80	7.03
initiation of PPI in the presence of DOAC	1.90	1.35	2.70	8.67	2.85	26.31
Use one drug in the hazard window and the other drug in the control window	2.46	1.06	5.74	NA*	NA*	NA*
**Apixaban**						
DOAC only	1.70	1.27	2.29	4.25	1.02	17.78
PPI only	1.55	1.41	1.70	14.49	10.44	20.11
both drugs were initiated together	1.74	1.01	3.01	19.00	1.35	267.03
initiation of DOAC in the presence of PPI	3.36	2.19	5.16	11.33	2.40	53.47
initiation of PPI in the presence of DOAC	2.05	1.38	3.04	17.00	2.61	110.78
Use one drug in the hazard window and the other drug in the control window	1.25	0.53	2.97	NA*	NA*	NA*

**Edoxaban**						
DOAC only	2.71	1.21	6.07	NA*	NA*	NA*
PPI only	1.57	1.44	1.72	15.06	10.88	20.84
both drugs were initiated together	1.50	0.28	7.91	1.00	0.03	38.26
initiation of DOAC in the presence of PPI	4.57	1.56	13.39	NA*	NA*	NA*
initiation of PPI in the presence of DOAC	1.83	0.50	6.78	NA*	NA*	NA*
Use one drug in the hazard window and the other drug in the control window	NA*	NA*	NA*	NA*	NA*	NA*

Abbreviation: OAC: oral anticoagulant, DOAC: direct anticoagulant, UK: United Kingdom, CPRD: Clinical Practice Research Datalink, HK: Hong Kong, CDARS: Clinical Data Analysis and Reporting System, CI: confidence interval, PPI: proton pump inhibitors, NA: not applicable
*No event or extreme small number of events on this parameter so no eligible estimate was shown.

Supplementary Table 8. Subgroup analysis by history of gastrointestinal bleeding for the association between concomitant use of OAC and PPI and gastrointestinal bleeding, compared with OAC only in cohort study

		UK CPRD				HK CDARS			
		**PS-HR**	**99% lower CI**	**99% Upper CI**	**Interaction p-value**	**PS-HR**	**99% lower CI**	**99% Upper CI**	**Interaction p-value**
**warfarin + PPI vs. warfarin alone**	**With history of GIB**	1.44	0.89	2.32	0.43	0.78	0.40	1.49	<0.01
	**Without history of GIB**	1.21	0.87	1.68		**4.19**	**2.44**	**7.18**	
**DOAC + PPI vs. DOAC alone**	**With history of GIB**	1.07	0.78	1.45	0.36	0.77	0.52	1.12	<0.01
	**Without history of GIB**	1.22	0.98	1.52		**3.69**	**2.57**	**5.31**	

Abbreviation: OAC: oral anticoagulant, PPI: proton pump inhibitors, DOAC: direct oral anticoagulant, GIB: gastrointestinal bleeding, UK: United Kingdom, CPRD: Clinical Practice Research Datalink, HK: Hong Kong, CDARS: Clinical Data Analysis and Reporting System, PS-HR: propensity score model hazard ratio, CI: confidence interval

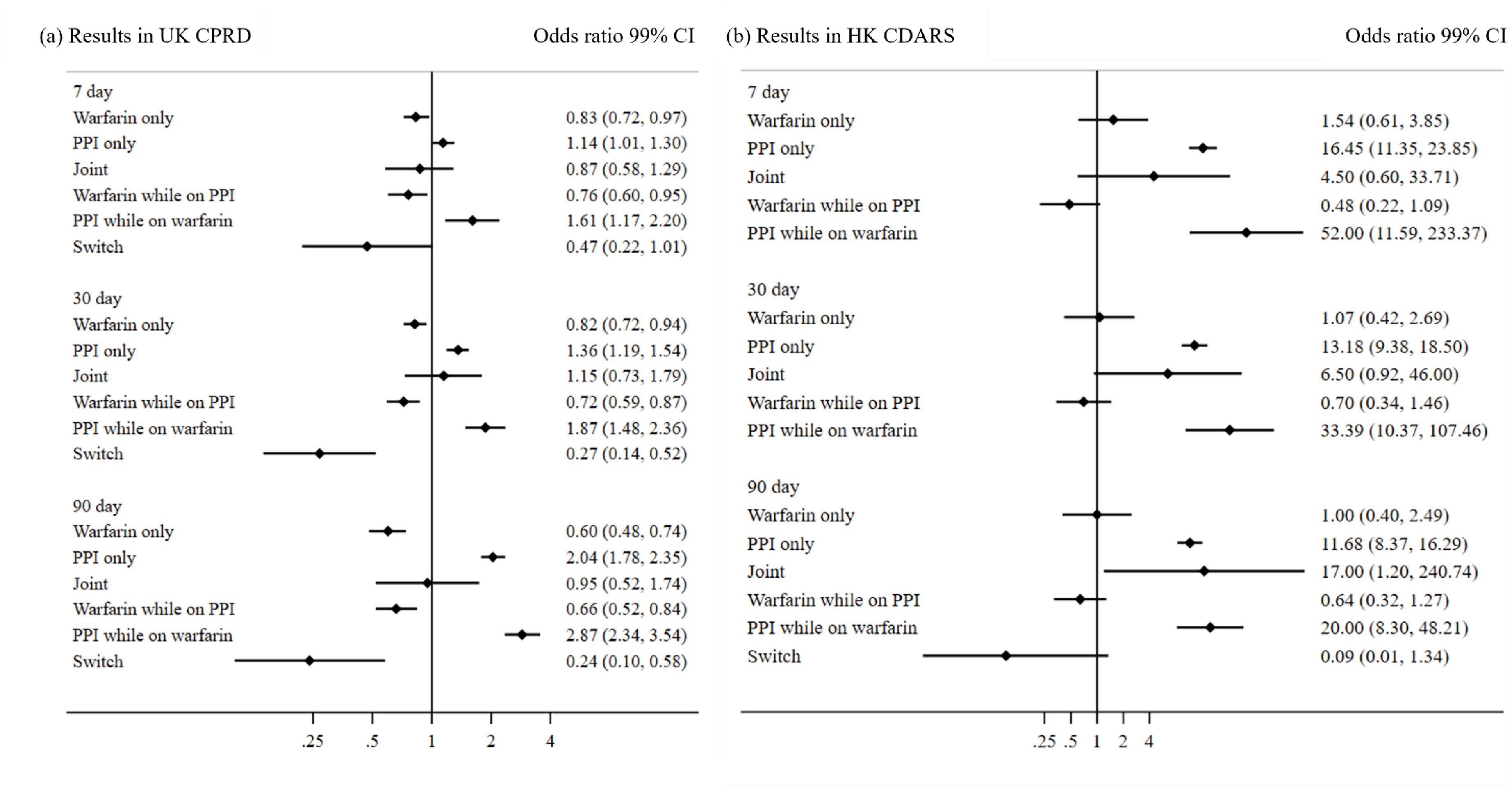

Supplementary Figure 1. Sensitivity analysis varying the length of hazard period for the association concomitant use of warfarin and PPI and gastrointestinal bleeding in case-crossover study

Abbreviation: UK: United Kingdom, CPRD: Clinical Practice Research Datalink, HK: Hong Kong, CDARS: Clinical Data Analysis and Reporting System, CI: confidence interval, PPI: proton pump inhibitors

Note: Parameter without eligible estimate was not shown in the figure which is because of no event or extreme small number of events.

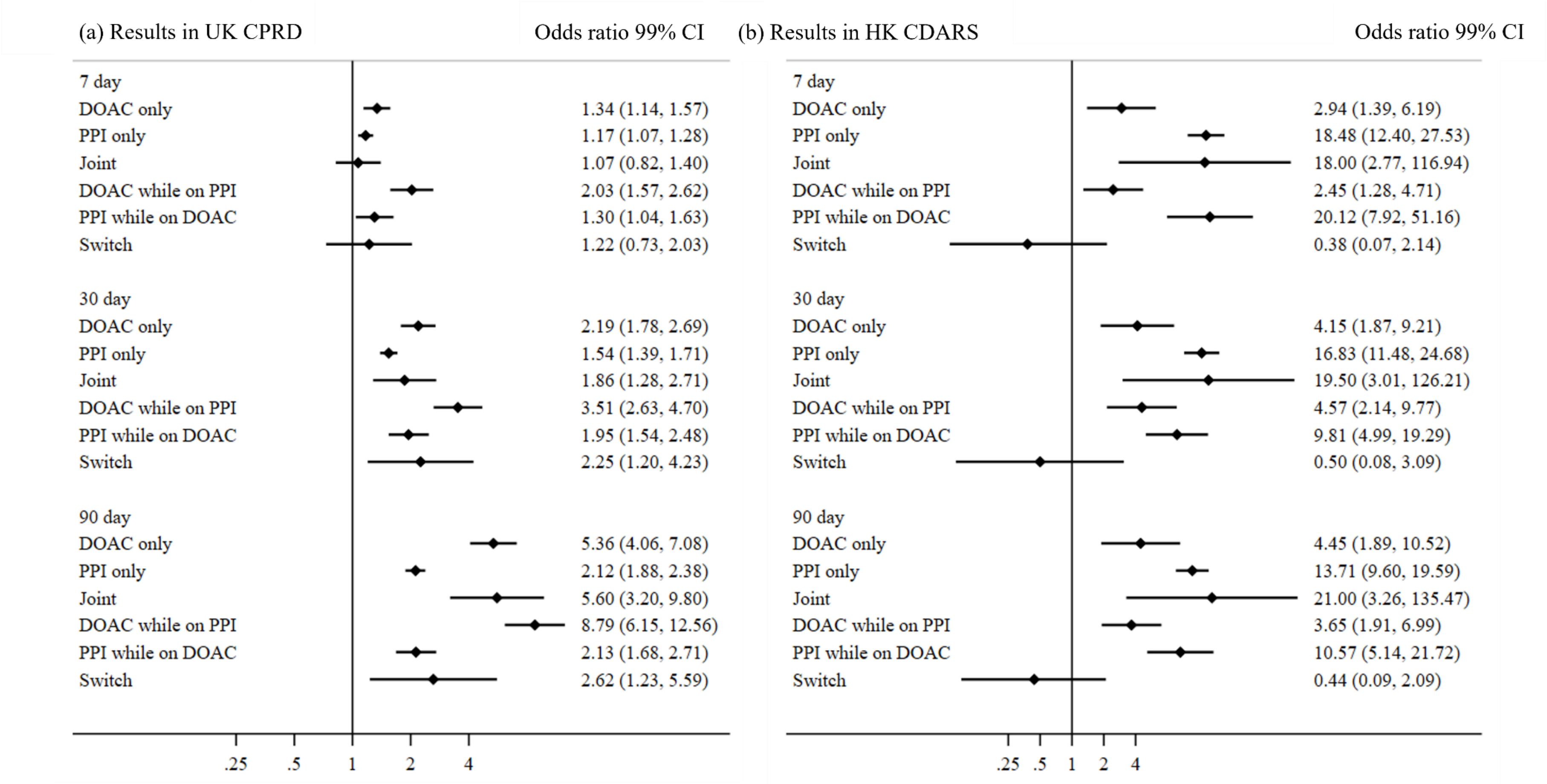

Supplementary Figure 2. Sensitivity analysis varying the length of hazard period for the association concomitant use of DOAC and PPI and gastrointestinal bleeding in case-crossover study

Abbreviation: UK: United Kingdom, CPRD: Clinical Practice Research Datalink, HK: Hong Kong, CDARS: Clinical Data Analysis and Reporting System, CI: confidence interval, DOAC: direct anticoagulant, PPI: proton pump inhibitors
Note: Parameter without eligible estimate was not shown in the figure which is because of no event or extreme small number of events.

A) Comparing warfarin + proton pump inhibitors with warfarin only

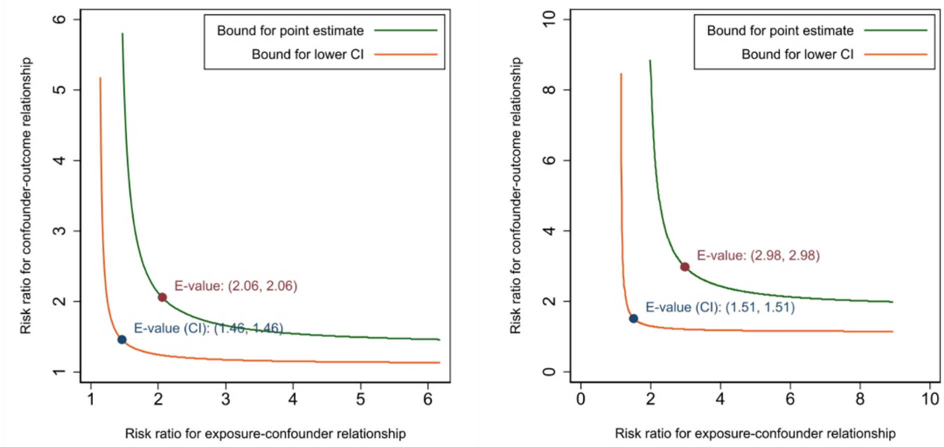

a) CPRD Aurum b) CDARS

B) Comparing direct oral anticoagulants + proton pump inhibitors with direct oral anticoagulants only

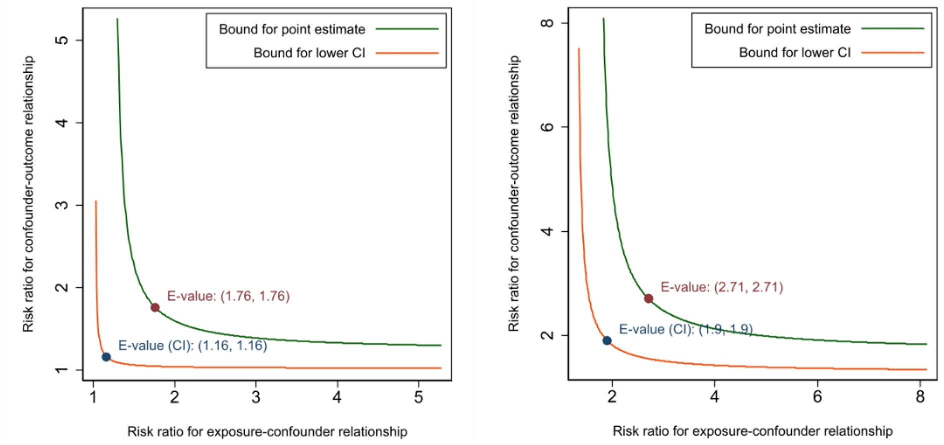

a) CPRD Aurum b) CDARS

Supplementary Figure 3. E-value for the lower 99% confidence interval and point estimate

Abbreviation: CPRD: Clinical Practice Research Datalink, CDARS: Clinical Data Analysis and Reporting System, CI: confidence interval

To potentially fully explain the propensity score weighted hazard ratio (PS-HR) or the lower bound of the 99% confidence interval (CI) in our study population, an unmeasured confounder would need to be associated (conditional on measured covariates) with either warfarin + proton pump inhibitor (PPI) or gastrointestinal bleeding (GIB) with a risk ratio of at least 2.06 (effect estimate) or 1.46 (lower bound) in Clinical Practice Research Datalink (CPRD) Aurum and 2.98 (effect estimate) or 1.51 (lower bound) in Clinical Data Analysis and Reporting System (CDARS).
To potentially fully explain the PS-HR or the lower bound of the 99%CI in our study population, an unmeasured confounder would need to be associated (conditional on measured covariates) with either direct oral anticoagulant + PPI or GIB with a risk ratio of at least 1.76 (effect estimate) or 1.16 (lower bound) in CPRD Aurum and 2.71 (effect estimate) or 1.90 (lower bound) in CDARS.
